# Editorial: Briefs in neural rehabilitation engineering

**DOI:** 10.3389/fresc.2024.1405549

**Published:** 2024-05-01

**Authors:** Ping Zhou, Raymond Kai-Yu Tong

**Affiliations:** ^1^ School of Rehabilitation Science and Engineering, University of Health and Rehabilitation Sciences, Qingdao, Shandong, China; ^2^ Department of Biomedical Engineering, The Chinese University of Hong Kong, Hong Kong SAR, Hong Kong, China

**Keywords:** neural engineering, rehabilitation, neurological conditions, briefs, perspectives

**Editorial on the Research Topic** Briefs in neural rehabilitation engineering

Neurological conditions can lead to important limitations in functioning and quality of life. Different deleterious functional changes may feature in a range of neurological conditions. Neural rehabilitation engineering integrates engineering principles and methods with neuroscience and rehabilitation medicine, leading to better health outcomes for neurological conditions. This Research Topic has collected brief research reports and perspective articles that cover a range of subjects in neural rehabilitation engineering.

While locked knee ankle foot orthosis (L-KAFO) has many short-term and long-term benefits for standing and walking of a patient with severe neuromusculoskeletal impairment of the lower limb, Ghoseiri and Zucker-Levin discussed the iatrogenic biomechanical and physiological perils of its long-term use. These include an increased risk of developing low back pain, osteoarthritis of the lower limbs and spinal joints, skin dermatitis, and ulceration, due to a range of musculoskeletal, integumentary, and functional changes. Future directions are discussed to reduce the negative consequences that arise from the long term use of L-KAFO.

Sachdeva et al. tested the efficacy of noninvasive spinal cord neuromodulation (SCiP™, SpineX Inc.) with activity-based neurorehabilitation therapy (ABNT) in improving the sensorimotor function of six children with cerebral palsy (CP). The combined therapy demonstrated a significant clinical improvement in sensorimotor function in the tested subjects within a short period (8 weeks) and was sustainable for an extended period (10 weeks). These preliminary findings support future large-scale randomized controlled trials to further evaluate this combined therapy. In addition, the underlying neurophysiological mechanisms require an in-depth investigation.

Zong et al. presented a radial extracorporeal shock wave therapy study for treating carpal tunnel syndrome (CTS). Sixteen CTS patients received radial extracorporeal shock wave therapy once a week for five consecutive weeks and demonstrated significantly improved outcome measurements. The findings confirmed the effectiveness of shock wave therapy for treating CTS symptoms and the associated sensory nerve property changes. A novel compound muscle action potential (CMAP) scan examination was applied to examine motor unit number and size alterations before and after the treatment, but yielded inconsistent results using different CMAP scan processing parameters. The reasons for this inconsistency are worthwhile targets for further investigation.

Telerehabilitation has emerged as a noteworthy complement to traditional rehabilitation services. Although there is accumulating evidence for the effectiveness of robot-assisted therapies for neural rehabilitation, the technologies are not widely adopted for home or tele-environments. Huang et al. discussed the technological readiness of personalized robots in the context of stroke telerehabilitation and identified potential challenges and barriers for their clinical translation. Future directions and strategies are also discussed to facilitate the integration of technological advances into standard clinical workflows, with a focus on promoting personalized robots for long-term telerehabilitation after stroke.

Echoing the perspective article by Huang et al., Qing et al. presented a study investigating the long-term effects of robot-assisted self-help telerehabilitation on upper limb motor function after stroke. Twenty-two patients with chronic stroke attended a 20-session telerehabilitation program assisted by the wrist/hand module of a mobile exoneuromusculoskeleton. The system integrated neuromuscular electrical stimulation and a robot into one mechatronic platform, driven by voluntary efforts. Significant improvement in the primary outcome measures was observed and maintained after 6 months, indicating the long-lasting effects of the robot-assisted telerehabilitation.

We hope that this Research Topic helps to promote a wider recognition of neural rehabilitation engineering, share the current state-of-the-art trends, challenges, and directions, and encourage further development of neural rehabilitation technologies.

